# New Opportunities for Modern Fungal Biology

**DOI:** 10.3389/ffunb.2020.596090

**Published:** 2020-09-10

**Authors:** Gustavo Henrique Goldman

**Affiliations:** Faculdade de Ciências Farmacêuticas de Ribeirão Preto, Universidade de São Paulo, São Paulo, Brazil

**Keywords:** filamentous fungi, yeasts, secondary metabolites, genome and evolution, biotechnology

The fungal kingdom is estimated to contain 2 to 4 million species yet less than 5% of them have been formally described due to under sampling in fungal diversity studies (Blackwell, 2011; Hawksworth and Lucking, 2017; James et al., 2020). Fungi originated from a flagellated ancestor diverging from their sister kingdom the animals ~1.3 billion years ago (Naranjo-Ortiz and Gabaldón, 2019; James et al., 2020). However, most of the current fungi grow as a simple multicellularity called the mycelium, a complex cytoplasmic network populated by nuclei. These organisms can have mycelial growth or unicellular growth (yeasts), or can switch from yeast-to-hypha-to-yeast depending on the extracellular stimuli. Some of the fungi can form very complex multicellular structures, such as complex fruiting bodies in the Basidiomycota. The fungal kingdom has been organized based on genome sequencing in several classes, such as Ascomycota, Basidiomycota, Zygomycota, Glomeromycota, Blastocladiomycota, and Chytridiomycota (for a more fully resolved, comprehensive fungal tree of life, please refer to James et al., 2020). Fungi are able to survive in a large spectrum of growth conditions and biological interactions, and thus have important economic impacts for humankind in many ways considering their influence on food losses through pre- and post-harvesting plant colonization, and animal diseases. They are also relevant human and animal pathogens, being recently responsible for the most significant waves of animal extinction, such as reptiles by Chytridiomycota species and bats, white-nose syndrome, by the ascomycete *Pseudogymnoascus destructans* (Fisher et al., 2012, 2020). Important calorie crops, such as wheat and soybean harvests are currently under threat from the wheat blast fungus, *Magnaporthe oryzae*, and the soybean rust fungus, *Phakopsora pachyrhizi* (Fisher et al., 2012). Forest species are in great danger because of plant fungal diseases, such as Dutch elm disease (*Ophiostoma novo-ulmi*) and chestnut blight (*Cryphonectria parasitica*) (Fisher et al., 2012). However, they can also have major beneficial interactions with plants and animals arising from symbiotic relationships, such as the Basiodiomycota mycorrhizae, plant-growth promoting fungi, such as the Ascomycete *Trichoderma* spp. (Guzmán-Guzmán et al., 2019) and ant fungal gardens (Moreau, 2020). Moreover, fungi are fundamental for biotechnological advancements, producing the vast majority of the industrial enzymes used in several different processes, and also in the production of organic acids, antibiotics, food and beverages (Meyer et al., 2016, 2020; Cairns et al., 2018; Wösten, 2019).

Fungal biology has emerged in the last 10 years as a very strong scientific field with a surge in publications and understanding of basic and applied biological processes. Traditionally, many fungal model systems have provided important biological discoveries that go from George Beadle's hypothesis “one gene, one enzyme” using *Neurospora crassa* as a model system (Beadle and Tatum, 1941) to the spectacular identification of essential components of the cytoskeleton, such as α-, β-, and γ-tubulin encoding genes in *Aspergillus nidulans* (Sheir-Neiss et al., 1978; Morris et al., 1979; Oakley and Oakley, 1989). This pioneering work has established the foundations for the introduction of molecular biology and genetical transformation systems in the ascomycetes *N. crassa* and *A. nidulans* (Case et al., 1979; Ballance et al., 1983), followed by the landmark genome sequencing of the *N. crassa* (Galagan et al., 2003) and *A. nidulans, A. oryzae*, and *A. fumigatus* (Galagan et al., 2005; Machida et al., 2005; Nierman et al., 2005). The pioneering work of Max Delbrück in the phototropism of the zygomycete *Phycomyces blakesleanus* opened many new frontiers for the understanding of sensing the environment by living organisms (Strauss, 2017). Genetic transformation systems and genome sequencing has expanded to a plethora of filamentous fungi making possible the progress in the understanding of specific ecological interactions with insects, mammals, and plants revealing highly sophisticated mechanisms of commensalism and pathogenesis, such as those observed for arbuscular mycorrhiza and fungal pathogens (Pradhan et al., 2019; Roth et al., 2019). The recent advances in Cas9 CRISPR technologies for filamentous fungi (Song et al., 2019) will provide amazing opportunities to understand several processes in fungi that are not easily amenable for genetic manipulation.

The ability of filamentous fungi to secrete a large array of enzymes able to degrade the most complex carbon and nitrogen substrates, and detoxify organic and inorganic matters, such as lignocellulosics (Bomble et al., 2017) and in the soil microbiomes upon climate change (Jansson and Hofmockel, 2020), confers a critical importance in the recycling processes of the biosphere. Fungi play pivotal roles in many microbial ecosystems yet, due their minimal presence in comparison to bacteria, their detection and analysis remains a challenge. This is being actively remedied by the improvement in genomic techniques which has allowed the increased detection of fungi in several microbiomes, including humans. Interesting and exciting hypotheses about their role in complex systems, such as their importance on human pancreatic cancer (Aykut et al., 2019), have thus emerged, broadening our perception of their influence. Interesting and unique fungal features were also revealed, such as the anaerobic gut fungi (Neocallimastigomycota) that have extreme genomes (over 80% A+T) and hydrogenosomes instead of mitochondria (Naranjo-Ortiz and Gabaldón, 2019).

I believe that the true strength of a field is determined by its top journals and their dedication to empowering researchers to pursue and highlight key research questions. *Frontiers in Fungal Biology* is a new venture within the field that will establish specialized hubs for all areas of research across fungal biology. This includes fungal genomics, evolution, physiology, metabolism, metabolites, mycotoxins, and biotechnology, expanding into studies focused on their presence in marine and freshwater environments, and their interactions with animals, plants, soils and the microbiome. These areas are grouped under dedicated specialty sections which are led and managed by reputable international researchers representing the most brilliant minds of the field. The journal structure will evolve in the coming years to meet the needs of our community and together, we will highlight the importance of fungal science in all aspects of our lives as we know it.

What are the examples of fungal science I would like to see published in our new journal?

1) Articles reporting new ideas, hypotheses, general commentaries, perspective, reviews, and opinions about several aspects of fungal biology. I would like to see our journal as a forum to debate the most exciting topics in the area.

2) Articles reporting fungi-plant and fungi-animal interactions, describing in more detail not only mechanisms of these interactions, but also how fungi benefit or combat other microbes in the same environment.

3) Articles reporting genomic studies but especially comparative genomics and general omics, discussing the possible implications of their findings in terms of fungal evolution, and how comparable and different fungi are from other organisms.

4) Articles reporting all the possible applications of fungal genetic traits and properties toward biotechnology, to serve humankind and improve our lives.

5) Articles reporting all the uniqueness and conservation of fungal physiology and metabolism mechanisms, taking advantage of the most modern technologies to characterize how fungi “see” the world and transduce this information in terms of hierarchical genetic programs and biochemical pathways.

6) Articles revealing all the richness of the production of secondary metabolites and toxins, and how fungi protect themselves against these molecules (self-protection).

7) Articles characterizing and investigating the splendorous biology and biodiversity of marine and freshwater fungi, especially their adaptation to extreme environments.

To conclude, I would like to stress my view that journal editors have the mission to help authors publish their research and boost their visibility to further the advancement of science. Maintaining a rigorous and collaborative reviewing process is a challenging task that falls onto authors, reviewers and editors, which is why keeping an open dialogue throughout the process can improve the scientific density of the manuscripts, their visibility to the scientific community, and thus the overall quality of the journal they are published in.

## Author Contributions

The author confirms being the sole contributor of this work and has approved it for publication.

## Conflict of Interest

The author declares that the research was conducted in the absence of any commercial or financial relationships that could be construed as a potential conflict of interest.
